# Influence of Foam Morphology on Flow and Heat Transport in a Random Packed Bed with Metallic Foam Pellets—An Investigation Using CFD

**DOI:** 10.3390/ma15113754

**Published:** 2022-05-24

**Authors:** Ginu R. George, Marina Bockelmann, Leonhard Schmalhorst, Didier Beton, Alexandra Gerstle, Andreas Lindermeir, Gregor D. Wehinger

**Affiliations:** 1 Institute of Chemical and Electrochemical Process Engineering, Clausthal University of Technology, Leibnizstr. 17, 38678 Clausthal-Zellerfeld, Germany; wehinger@icvt.tu-clasuthal.de; 2 CUTEC Research Centre, Clausthal University of Technology, Leibnizstr. 23, 38678 Clausthal-Zellerfeld, Germany; marina.bockelmann@cutec.de (M.B.); andreas.lindermeir@cutec.de (A.L.); 3 Alantum Europe GmbH, Balanstr. 73, Building 21 A, 81541 Munich, Germany; lschmalhorst@alantum.com (L.S.); dbeton@alantum.com (D.B.); agerstle@alantum.com (A.G.)

**Keywords:** metallic foam, CFD, fixed-bed reactor, friction factor, heat transfer coefficient

## Abstract

Open-cell metallic foams used as catalyst supports exhibit excellent transport properties. In this work, a unique application of metallic foam, as pelletized catalyst in a packed bed reactor, is examined. By using a wall-segment Computational Fluid Dynamics (CFD) setup, parametric analyses are carried out to investigate the influence of foam morphologies (cell size ϕ=0.45–3 mm and porosity ε=0.55–0.95) and intrinsic conductivity on flow and heat transport characteristics in a slender packed bed $\left(N=D/d_{p}=6.78\right)$ made of cylindrical metallic foam pellets. The transport processes have been modeled using an extended version of conventional particle-resolved CFD, i.e., flow and energy in inter-particle spaces are fully resolved, whereas the porous-media model is used for the effective transport processes inside highly-porous foam pellets. Simulation inputs include the processing parameters relevant to Steam Methane Reforming (SMR), analyzed for low ($Re_{p}\sim 100)$ and high ($Re_{p}\sim 5000)$ flow regimes. The effect of foam morphologies on packed beds has shown that the desired requirements contradict each other, i.e., an increase in cell size and porosity favors the reduction in pressure drop, but, it reduces the heat transfer efficiency. A design study is also conducted to find the optimum foam morphology of a cylindrical foam pellet at a higher $Re_{p}\sim 5000$, which yields ϕ = 0.45, ε = 0.8. Suitable correlations to predict the friction factor and the overall heat transfer coefficient in a foam-packed bed have been presented, which consider the effect of different foam morphologies over a range of particle Reynolds number, $100\leq Re_{p}\leq 5000$.

## 1. Introduction

**Figure 1:**
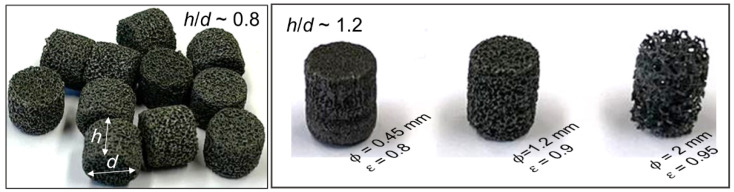
Metallic foam pellets—different sizes and morphology: cell size (𝜙) and porosity (*ε*).

Open-cell metallic foams are regarded as a versatile engineering material, as they can be used in various applications including heat exchangers, energy absorbers, filters, porous electrodes, fluid mixers, and so on [1,2,3]. Their unique properties, such as high porosity (75–95%), high surface area (up to 10,000 m^2^/m^3^), high intrinsic solid conductivity, and rigorous surface texture, have made them an excellent choice for catalyst supports [4]. As a structured catalyst support, foam monoliths have been shown to have high heat and mass transfer capabilities [5,6]. This motivates the development of pelletized metallic foams [7] for application in fixed-bed catalytic processes, to create randomly packed beds as shown in Figure 1. Having a high mechanical strength allows the metallic foam pellets to load and unload easily into multi-tubular reactors of more than 8 m in length without breaking. Furthermore, an innovative powder metallurgical process has been identified for the production of alloyed foam pellets (e.g., NiCrAl, FeCrAl) which could maintain structural stability even at medium-high temperatures (up to 1000 °C) [8]. It is believed that metallic foams in pellet shape are a breakthrough in catalyst substrates since the conventional ceramic catalyst pellets are unable to meet the entire design requirements of a fixed-bed reactor such as lower pressure drop and high heat transfer [9].

Slender packed bed reactors ($D/d_{p}\leq 10$) are the preferred reactor type for an efficient removal or addition of heat, i.e., for highly exothermic or endothermic catalytic reactions. They have complex transport characteristics due to local bed structure effects [10], different flow regimes, and their interaction between different heat transfer mechanisms [11]. In addition to shape and size, the pelletized foams offer extra design flexibility due to their morphologies, especially cell size and porosity, as shown in Figure 1. Thus, from a manufacturing perspective, it is imperative to find out the optimal foam morphology, as well as shape and size in terms of their transport behavior, prior to producing foam pellets in large quantities. Several experimental [12,13] and numerical studies [14,15,16] have been reported for foam monoliths or structural types; however, literature on the pelletized metallic foam in randomly packed beds is scarce. Kolaczkowski et al. [17] carried out experiments in a slender packed bed made of cubic foam pellets to investigate the pressure drop and thermal performance. Their experiment showed a significant reduction in pressure drop in comparison to one-hole ceramic pellets, while the heat transfer performance was comparable; they recommended exploring different shapes and morphologies. It is indeed time-consuming and expensive to rely solely on experimental studies, especially while exploring an expansive design space: foam shapes, sizes, morphological parameters, and operating conditions relevant to a particular chemical process. A reliable simulation tool is needed to support and accelerate the development of pelletized foams.

To simulate the transport processes in slender packed beds, Particle-resolved Computational Fluid Dynamics (PRCFD) has been extensively used, which takes into account the actual packed bed geometry, thereby solving the flow along interstitial voids [18,19]. However, applying such a detailed PRCFD approach in a foam-packed bed is computationally intensive, when resolving flow through the fine inner geometries of foam pellets, i.e., on the strut level. In prior works [20,21], we have introduced a modified version of PRCFD, in which the foam pellets are considered as porous media, and appropriate closure equations are used to account for the pressure loss and energy transport inside the pellets, i.e., flow along inter-particle spaces are considered as they are, whereas inner foam geometric features are not physically resolved. The Rigid Body Dynamics (RBD) method was used to generate the packing structure. The CFD model has been validated with experimental data for pressure drop and axial bed temperature, with excellent agreement [20,21]. Although the proposed PRCFD workflow can reduce the computation effort relatively, it is not very efficient for simulations like parametric analysis, since it takes from several hours to days for a bed containing more than 1000 particles (computed by 1 CPU—intel Core i7-8700K). For such simulation purposes, a wall-segment model with shorter bed geometry is used within the PRCFD framework. Dixon et al. [22] have used a 120° pellets containing wall-segment CFD setup to investigate flow, temperature distribution, and reaction. They analyzed the influence of different hole numbers on a cylinder particle and concluded that a six-hole cylinder allows for better temperature distribution and reaction in SMR processing conditions. Wehinger et al. [23] have used a 45° sliced bed segment composed of spherical particles to model Dry Reforming of Methane (DRM) over nickel catalyst, with microkinetics describing the surface reactions. The CFD simulation without chemical reaction was able to produce the same axial bed temperature profile that was observed experimentally, whereas the original microkinetics formulation had to be modified to match with DRM experimental data due to thermodynamic inconsistencies in the used kinetics model. The results of these previous studies are encouraging for the use of wall-segment PRCFD.

In this work, a wall-segment PRCFD setup realized with a 90° sliced bed geometry is used to investigate the influence of foam morphologies—cell size and porosity—on flow and heat transfer within a packed bed made of cylindrical foam pellets. The PRCFD modeling strategy used here is the same one that was developed for a full-bed structure, i.e., porous media at the individual foam pellet level. The effect of foam thermal conductivity on radial heat transport is also analyzed. The simulation is carried out for the processing conditions relevant to SMR on an industrial scale. A design study is also carried out to find the optimum foam pellet morphology. By using CFD data, suitable correlations have been derived for the friction factor and the heat transfer coefficient of a foam-packed bed, accounting for different foam morphologies and particle Reynolds number.

## 2. Materials and Methods

### 2.1. Study Configuration

A slender tubular packed bed ($D/d_{p}=6.78)$ composed of cylindrical alloyed foam pellets, NiCrAl (71% Ni, 19% Cr, 10% Al), was considered. Table 1 provides important properties of the foam pellet. The flow regimes in the packed bed were defined by particle Reynolds number $Re_{p}=\rho v_{s}d_{p,v}/\mu$, where ρ is the density, $v_{s}$ is the superficial velocity and μ is the dynamic viscosity of the fluid medium. The diameter of a sphere of equivalent volume $d_{p,v}={\left(6V_{p}/\pi \right)}^{1/3}$ was chosen as the characteristic length, with Vp=π4dp2h as the apparent particle volume, i.e., inner porosity ε of the foam pellet was not considered. Here, $d_{p}$ and h are the diameter and the height of the pellet.

To analyze the influence of foam morphologies on flow and heat transfer within metallic foam-packed bed, the bed friction factor $f^{*}$ and the overall heat transfer coefficient U were calculated according to Equations (1)–(4), for different cases: (1) Varying cell sizes ϕ (0.45–3 mm) at constant porosity; (2) Changing porosities ε (0.55–0.95) keeping same ϕ; (3) different intrinsic foam conductivities at constant ϕ and ε. As a base case, ϕ = 1.2 mm and ε = 0.9 was chosen. Moreover, each case was analyzed for $Re_{p}$~100 and $Re_{p}$~5000, since the effect of foam morphology on transport processes is also dependent on flow regimes. It should be noted that the bed structural properties such as mean interstitial bed voidage and particle orientation are kept constant for all the cases.

The friction factor $f^{*}$ is given by:

(1) $$f^{*}=\frac{\Delta P}{L}\frac{d_{p,v}}{\rho v_{s}^{2}}\frac{\xi^{3}}{1-\xi }$$

where: ΔP/L is the specific pressure drop along a bed height *L*, ξ is mean interstitial bed voidage without considering the porosity of foam pellets.

The overall heat transfer coefficient *U* can be defined as:

(2) $$U=\frac{Q}{A^{\prime }\Delta T_{LM}}$$

Here, *Q* is the total heat transfer rate, $A^{\prime }$ is the area available for heat transfer, and $\Delta T_{LM}$ is the logarithmic mean temperature difference, computed as per Equation (3) for a constant wall temperature $T_{w}$ with mixed mean fluid temperatures at the inlet $T_{\mathrm{in}}$ and the outlet $T_{\mathrm{out}}$ [24].

(3) $$\Delta T_{\mathrm{LM}}=\left(T_{\mathrm{out}\;}-T_{\mathrm{in}\;}\right)/\log\left[\left(T_{w}-T_{\mathrm{in}\;}\right)/\left(T_{w\;}-T_{\mathrm{out}\;}\right)\right]$$

Equation (2) can be normalized by combining with $d_{p,v}$ and fluid conductivity $\lambda_{f}$:

(4) $$U^{*}=\frac{Ud_{p,v}}{\lambda_{f}}$$

### 2.2. Particle-Resolved CFD

#### 2.2.1. Packed Bed Geometry

**Figure 2:**
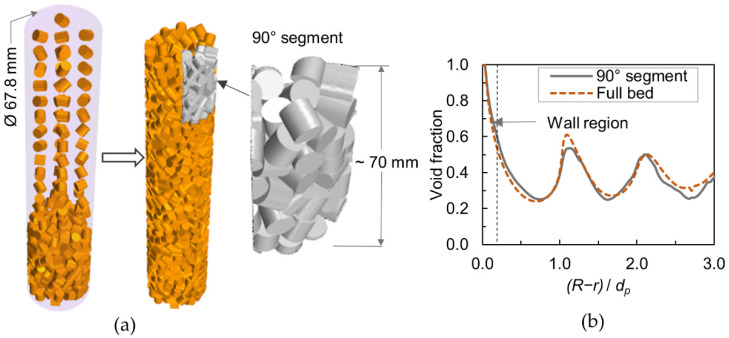
(**a**) Random bed generation and 90° segmented bed; (**b**) Averaged radial void fraction (Here, the particle inner porosity is not considered).

The bed geometry was created by the open-source software Blender, in which the physical effects occurring while pouring catalyst pellets into a reactor tube can be simulated by the Rigid Body Dynamics (RBD) approach supported by the Bullet physics library [25]. The application of Blender in packing generation has been discussed in many recent works [26,27,28]. In a previous work [20], we have verified the impact of RBD parameters such as friction factor and restitution coefficient as well as different catalyst loading methods on the generated packing structures. Blender settings identified as suitable for creating a packing of cylindrical particles were used in this study. As illustrated in Figure 2a, the particles aligned in a random array, fell freely into the container and settled to form a randomly packed bed. For the wall-segment CFD set-up, a 90° bed sector with a height of about 70 mm was sliced from the full bed, see Figure 2a.

The slender packed beds, $D/d_{p}\leq 10$, exhibit a significant variation in radial void fraction due to the influence of reactor wall, which strongly affects the transport characteristics. Figure 2b shows the comparison of azimuthally averaged radial voidage between the 90° bed segment and the full bed. The agreement is good, except for slight differences in the first peak and towards the bed center. These variations can be expected in such a short bed segment. The mean squared error between the void fraction profiles is about 0.16%, indicating that the bed segment is sufficient to reproduce the transport behavior of a full bed with reasonable accuracy.

#### 2.2.2. Model Equations

The conservation equations for mass, momentum, energy and species transport were considered in a three-dimensional domain for the laminar ($Re_{p}$~100) and turbulent ($Re_{p}$~5000) flows, the latter using Reynolds averaged Navier-Stokes (RANS) approach. The turbulence was modeled by Realizable K-epsilon with Two-layer All *y*^+^ wall treatment [29], which is preferred for PRCFD simulations [18]. The governing equations over a finite volume at steady-state are briefly reviewed here; a detailed description can be found elsewhere [30].

Continuity equation:

(5) $$\oint_{A}\rho v\cdot da=\int_{V}S_{u}dV$$

where: v is the velocity, a is the area vector, $S_{u}$ is source term, V is volume.

Momentum equation:

(6) $$\oint_{A}\rho v⊗v\cdot da=-\oint_{A}pI\cdot da+\oint_{A}T\cdot da+\int_{V}f_{b}dV+\int_{V}S_{u}dV$$

where: ⊗ denotes the outer product, p is pressure, T is the viscous stress tensor, I is the Identity tensor, $f_{b}$ is the resultant of body forces.

Energy equation:

(7) $$\oint_{A}\rho Hv\cdot da=-\underset{\mathrm{Conduction}\;}{\underbrace{\oint_{A}{\dot{q}}^{\prime \prime }\cdot da}}+\underset{\mathrm{Viscous}\;\mathrm{Work}\;}{\underbrace{\oint_{A}T\cdot vda}}+\int_{V}f_{b}\cdot vdV+\int_{V}S_{u}dV$$

where: H is total enthalpy, ${\dot{q}}^{\prime \prime }$ is the heat flux vector.

The viscous stress tensor for a Newtonian fluid is given by T=2μD−23μ(∇⋅v)I, where μ is the dynamic viscosity of the fluid and the rate of deformation tensor D=12(∇v+(∇v)T). The local heat flux in terms of thermal conductivity λ and temperature gradient ∇T is ${\dot{q}}^{\prime \prime }=-\lambda \nabla T$. The energy transfer across the contact interface between different mediums was modeled by the conjugate heat transfer approach [31].

Species transport:

For a multi-component gas mixture, the transport equation for a component species i is given by:

(8) $$\oint_{A}\left(\rho Y_{i}v\right)\cdot da=\oint_{A}\;J_{i}\cdot da+\int_{V}S_{Y_{i}}dV$$

where, $Y_{i}=m_{i}/m$ is the mass fraction of species i, with mass $m_{i}$ and total mixture mass m. The molecular diffusive flux $J_{i}$ based on mixture-average formulation is:

(9) $$j_{i}=-\rho \frac{Y_{i}}{X_{i}}D_{i}^{M}\nabla X_{i}$$

The effective diffusion coefficient $D_{i}^{M}$ of the species i with other mixture components is given by:

(10) $$D_{i}^{M}=\frac{1-Y_{i}}{\sum_{j\neq i}^{N_{G}}X_{j}/D_{ij}}\;\mathrm{for}\;i=1,\ldots ,N_{G}$$

where: $N_{G}$ is the number of species, $X_{i}$ is the molar fraction given by Equation (11) with molecular weight $M_{i}$.

(11) $$X_{i}=\frac{1}{\sum_{j=1}^{N_{g}}\frac{Y_{j}}{M_{j}}}\frac{Y_{i}}{M_{i}}$$

For modeling turbulent flow by the RANS approach, the scalar quantities in the above-mentioned equations are decomposed into a time-averaged value and a fluctuating component. A general scalar transport Θ as per RANS approach is represented as:

(12) $$\Theta \left(x_{i},t\right)=\bar{\Theta }\left(x_{i}\right)+\Theta^{\prime }\left(x_{i},t\right)$$

(13) $$\nabla \cdot \left(\rho \bar{v}\bar{\Theta }\right)=\nabla \cdot \left(\Gamma \nabla \Theta +\Gamma_{t}\nabla \Theta \right)$$

where: $\bar{\Theta }$ is the time-averaged and $\Theta^{\prime }$ is the fluctuating component; Γ is the general diffusion coefficient and $\Gamma_{t}$ is the turbulent diffusion coefficient. The turbulence modeling is an elaborative topic and has been explained in many fundamental books [32].

Coupling intra-particle transport processes with surrounding fluid:

The critical part of modeling a foam-packed bed is to reliably define the transport processes inside the porous foam pellets. By using a porous-media approach at the individual foam pellet level, a workflow that requires relatively less computation effort has been developed, see [20,21]. Thus, the fine inner structures were not spatially resolved; rather closure equations were used to account for the pressure loss and the effective thermal conductivity of foam pellets.

The momentum loss inside the pellets was considered by the Lacroix correlation [33], which is a modified form of the classical Ergun equation [34]. By cubic-cell geometric similarity, the equivalent particle diameter term in the original Ergun equation is re-formulated according to foam structural parameters such as porosity ε, cell size ϕ, pore diameter a, and strut diameter $d_{s}$, see Equations (14,15). The pore diameter is approximated from the cell size as a=ϕ/2.3, see details of the derivation in [33]. The viscous and inertial terms in Equation (14) consist of Ergun constants A = 150 and B = 1.75.

(14) $$\frac{\nabla p}{L}=A\frac{{\left(1-\varepsilon \right)}^{2}\mu }{\varepsilon^{3}{\left(1.5d_{s}\right)}^{2}}v_{s}+B\frac{\left(1-\varepsilon \right)\rho }{\varepsilon^{3}\left(1.5d_{s}\right)}v_{s}^{2}$$

(15) $$d_{s}=\frac{a{\left[\left(\frac{4}{3\pi }\right)\left(1-\varepsilon \right)\right]}^{\frac{1}{2}}}{1-a{\left[\left(\frac{4}{3\pi }\right)\left(1-\varepsilon \right)\right]}^{\frac{1}{2}}}$$

The Lacroix correlation is primarily formulated for a monolith-type of foam arrangement within a reactor tube, in which the entire flow path passes through the foam structure. In a randomly packed bed setup, however, flow occurs through the foam pellets as well as around them. In order to apply to a packed bed arrangement, the original Lacroix equation Equation (14) has to be slightly modified by including a correction term for porosity ($\varepsilon^{\prime }=0.977\cdot \varepsilon$). This correction term has been verified by pressure drop experiments and corresponding PRCFD simulations, see [20].

The thermal transport inside the foam pellets was modeled by the thermal-equilibrium approach, which disregards the temperature difference between fluid and solid phases; such an assumption simplifies the modeling effort and reduces the computational time. The corresponding energy equation is:

(16) $$\nabla \cdot \left(\varepsilon \rho_{\mathrm{fluid}\;}H_{\mathrm{fluid}\;}v\right)=\nabla \cdot \left(\lambda_{\mathrm{eff}\;}\nabla T_{\mathrm{fluid}\;}\right)+\nabla \cdot \left(\varepsilon T\cdot v\right)$$

where: v is the physical velocity, $H_{\mathrm{fluid}}$ is the total enthalpy of the fluid, $\rho_{\mathrm{fluid}}$ is the fluid density.

The effectively thermal conductivity $\lambda_{\mathrm{eff}\;}$ is usually formulated by the combination of fluid conductivity $\lambda_{\mathrm{fluid}}$ and foam bulk conductivity $\lambda_{\mathrm{foam},b}$, weighted by the porosity. Several models based on foam monoliths have been presented in the literature—see a comprehensive review article by Ranut [35]. In prior work [21], we have verified the applicability of an effective conductivity model proposed by Schuetz and Glicksman [36] Equation (17), for the randomly packed foam bed. The comparison with experimental data has shown a very good agreement in terms of axial bed temperature, see [21]

(17) $$\lambda_{\mathrm{eff},\mathrm{SG}}=\varepsilon \lambda_{\mathrm{fluid}}+\left(1-\varepsilon \right)\frac{1}{3}\lambda_{\mathrm{foam},b}$$

The bulk foam conductivity $\lambda_{\mathrm{foam},b}$ for a Nickel-Chromium alloy as a function of temperature T is given by [37]:

(18) $$\lambda_{\mathrm{foam},b}=5.192+0.0192\times T\;\mathrm{for}\;0\;{}^{\circ}C<T<1200\;{}^{\circ}C$$

#### 2.2.3. Computational Domain, Boundary Conditions, and Solving

**Figure 3:**
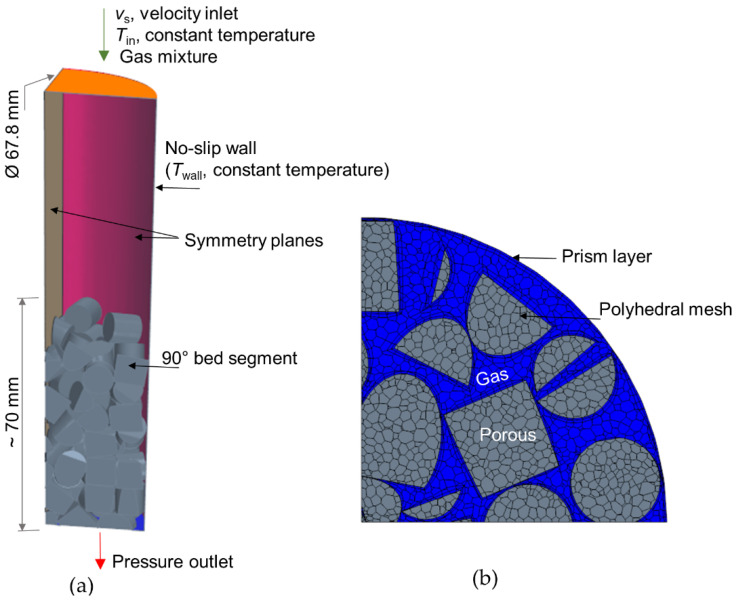
(**a**) Simulation setup overview; (**b**) Mesh types (view on a bed cross-section).

Figure 3a illustrates the numerical setup, realized with a 90° segmented bed structure enclosed by a corresponding tube segment. Accordingly, the computational domain consists of gas-phase and porous regions, which correspond to inter-particle voids and foam pellets, respectively. The meshing process was carried out by Siemens Simcenter STAR-CCM+ software with polyhedral type of cells in the bulk region and prism-layer cells in the solid-gas interfaces, i.e., near the reactor wall and around the pellet peripheries, see Figure 3b. To ensure the mesh quality at particle-particle contact regions, the contact modification methods presented in ref. [38,39] were followed. The mesh quality, mainly of thin gas-phase cells between the particles, is critical for achieving better convergence of solution variables. However, in the case of a foam-packed bed, this is not pertinent, since the possibility for a sharp boundary layer surrounding the foam particles is very low due to intra-particle flow. The entry portion of the simulation domain was extended with gas-phase cells to minimize the inlet effects, see Figure 3a. After several consecutive mesh refinements, the cell count of about 156,000 was finalized, upon which the solution variables have shown negligible difference with further grid refinement.

The boundary conditions are schematically represented in Figure 3a. At the inlet to the system, Dirichlet boundary types of specified velocity, temperature, and feed compositions were imposed as listed in Table 2. Two different velocities of 0.032 and 1.62 m s^−1^ were used, which correspond to $Re_{p}$~100 and ~5000, respectively. The mixture compositions and operating temperatures shown in Table 2 are relevant to SMR [40]. A no-slip boundary condition was assigned at the reactor wall with a constant wall temperature of 1000 K and a symmetry wall was set for the wall portions corresponding to the cut segment, see Figure 3a. At the reactor exit, the pressure outlet boundary condition was used. A working pressure of 29 bar was considered, which is typical for SMR under industrial conditions [41]. The ideal gas law was assumed to determine the density of mixture components according to the variation in temperature and pressure. The dynamic viscosity was based on the Chapman-Enskog model and the thermal conductivity of the gas via kinetic theory [29].

Siemens Simcenter STAR-CCM+ 15.02 was used to carry out CFD simulations, with the finite-volume method to solve the conservation equations. The closure equations presented in Section 2.2.2 were formulated within the software as field functions. The segregated flow and energy solvers with the Algebraic Multigrid (AMG) iterative method [29] were used to conduct steady-state simulations. An upwind differencing scheme of second-order accuracy was used for the discretization of convective and diffusion terms. The convergence criteria were monitored on solution variables such as velocity, pressure, and temperature at the outlet boundary and at several distinct point probes.

## 3. Results and Discussion

### 3.1. Validation with Experimental Data

It is important to make sure that the wall-segment CFD setup is reliable in capturing the flow characteristics and heat transport similar to those of a full bed. The experimental data and detailed simulation results from prior works [20,21] are used for the validation. It should be noted that the CFD simulation for the purpose is carried out according to the experiment setup, i.e., nitrogen gas, cylindrical foam pellet system, with foam properties: ϕ = 1.2 mm and ε = 0.9.

**Figure 4:**
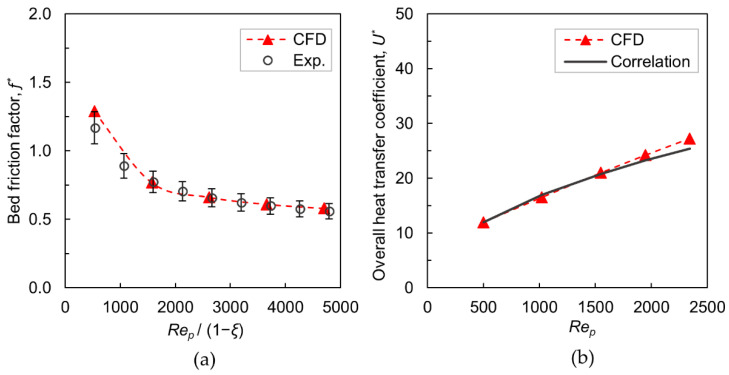
(**a**) Bed friction factor versus modified particle Reynolds number (𝜉 = ~0.40 is the mean interstitial bed voidage, experiment data from ref. [20] added with error bars of ±10%); (**b**) Normalized overall heat transfer coefficient versus particle Reynolds number (correlation is Equation (19) from ref. [21]).

The bed friction factor provided by Equation (1) is used to evaluate the hydrodynamic characteristics. Figure 4a depicts the comparison of friction factor obtained by the wall-segment CFD simulations with experimental data measured in a real bed structure. A good agreement is observed over the investigated range of $Re_{p}$, and deviations are within ±10%, which is acceptable. To quantify thermal transport behavior, the normalized overall heat transfer coefficient $U^{*}$ is determined from the heat transfer simulations by using Equations (2)–(4). Figure 4b shows the comparison of $U^{*}$ computed with the predicted values by a correlation, Equation (19), which has been formulated based on heat transfer experiments in a foam-packed bed, see [21]. The agreement is promising, as the differences are very minimal.

(19) $$U^{*}=0.586Re_{p}^{0.486}$$

The validation outcomes clearly demonstrate that the adopted wall-segment PRCFD setup is suitable for evaluating the transport properties of metallic foam-packed beds qualitatively, with reasonable accuracy.

### 3.2. Effect of Cell Size

**Figure 5:**
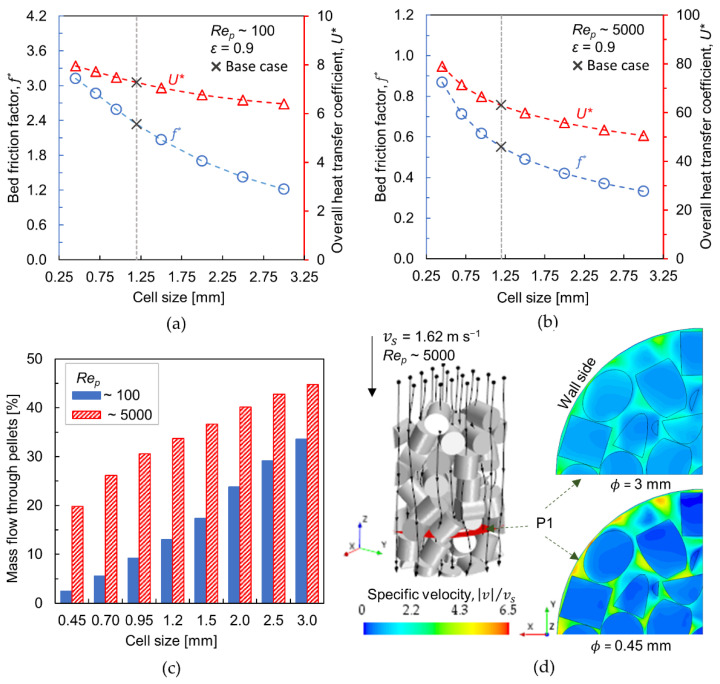
(**a**,**b**) Bed friction factor (left *y*-axis, circle markers) and overall heat transfer coefficient (right *y*-axis, triangle markers) versus cell size (𝜙) at *Re_p_*~100 and 5000, respectively (Base case denotes, 𝜙 = 1.2 mm); (**c**) Percentage of mass flow through the foam pellets; (**d**) Normalized velocity contours at *Re_p_*~5000 for cell sizes 3 mm and 0.45 mm (Velocity contours are displayed along the cross-sectional plane P1, Black lines running through the bed illustrate stream lines).

The influence of cell size on transport characteristics has been investigated by considering different cell sizes between 0.45 and 3 mm at constant porosity (ε=0.9). Figure 5a,b show the bed friction factor and overall heat transfer coefficient for $Re_{p}$~100 and $Re_{p}$~5000, respectively. As the cell size increases, the friction factor and overall heat transfer coefficient decrease. The reduction in pressure drop with increased cell size has been verified even in foam monoliths [12,33]. At $Re_{p}$~100 and comparing with base case: ϕ = 1.2 mm, the relative change in friction factor is about +34% when lowering the cell size to 0.45 mm, whereas it is −48% upon increasing the cell size to 3 mm. The respective values at $Re_{p}$~5000 are +58% and −40%. In a similar manner, the relative change in overall heat transfer coefficient by changing the cell size from 0.45 mm to 3 mm is +9% and −12% at $Re_{p}$~100 while it is +25% and −20% at $Re_{p}$~5000. It is evident that the variation in cell size causes more noticeable effects in friction factor than that of heat transfer coefficient. Also, the influence seems relatively higher at $Re_{p}$~5000.

The most distinctive feature of a foam-packed bed is an additional flow path through the highly porous foam pellets. Therefore, the transport characteristics of such packed beds are strongly influenced by the amount of flow passing through the pellets. A qualitative estimation of the amount of intra-particle flow is shown in Figure 5c, as the percentage of total mass flow, quantified along a cross-sectional plane—P1 (see Figure 5d). It is worthwhile to point out that the variations in local bed voidage and particle orientation might cause differences in mass flow through the pellets at different cross-sectional planes by about ±5%—more details in ref. [20]. From Figure 5c, it is clear that the larger cells combined with a higher flow velocity favor the flow through the foam pellet. For $Re_{p}$~5000 and cell size 3 mm, the foam pellets convey about 45% of the total mass flow, whereas the amount reduces to 34% when $Re_{p}$~100. At higher flow rates, the inertial force dominates the pellet’s flow resistance via Equation (14), thereby allowing an increased amount of intra-particle flow. Thus, a significant reduction in pressure drop can be achieved using larger foam cells, especially at higher flow rates.

The increased cell size, however, does not favor radial heat transport, and the negative impact is more obvious at higher $Re_{p}$~5000. When the flow rate is higher, convection is the predominant heat transfer mechanism (see Section 3.4), which is enhanced by lateral-mixing of the fluid around the particles in addition to localized turbulence. Indeed, the internal flow conveyed by the foam pellets hinders the intensity of fluid mixing. Figure 5d shows the normalized velocity contours along a cross-sectional plane (P1) for different cell sizes: 0.45 and 3 mm, at $Re_{p}$~5000. The reduced cell size results in an increased flow resistance at the pellet level. Consequently, the flow is diverted around the pellets with enhanced interstitial velocity. At $v_{s}$ = 1.62 m s^−1^, the localized interstitial velocity rises by a factor of about 6 for ϕ = 0.45 mm, whereas it decreases to 3.5 for ϕ = 3 mm. From a design perspective, it is therefore important to select an optimum cell size that yields a balance between the pressure drop and heat transfer efficiency.

### 3.3. Effect of Porosity

**Figure 6:**
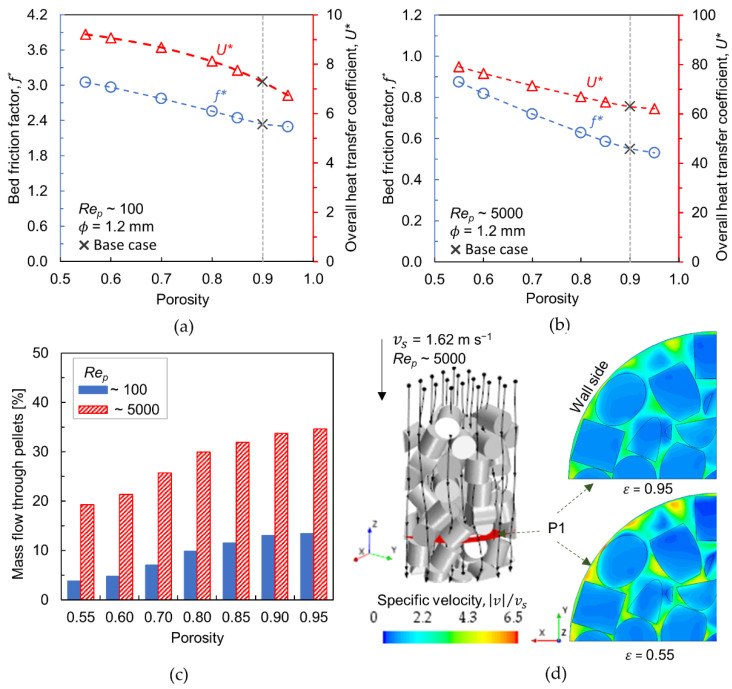
(**a**,**b**) Bed friction factor (left *y*-axis, circle markers) and overall heat transfer coefficient (right *y*-axis, triangle markers) versus porosity at *Re_p_*~100 and 5000, respectively (base case denotes *ε* = 0.9); (**c**) Mass flow through the foam pellets; (**d**) Normalized velocity contours at *Re_p_*~5000 for porosities 0.55 and 0.95 (Velocity contours are displayed along the cross-sectional plane P1, Black lines running through the bed illustrate stream lines).

The sensitivity of the pellet porosity towards transport characteristics has been examined by varying the porosity: 0.55–0.95 and keeping cell size constant (ϕ = 1.2 mm). Figure 6a,b depict the bed friction factor and overall heat transfer coefficient for $Re_{p}$~100 and $Re_{p}$~5000, respectively. As observed for the cell size, the friction factor and the overall heat transfer coefficient decrease with the increase in porosity. A similar trend in the case of friction factor has been reported in ref. [42]. By reducing the porosity to 0.55 and comparing it with base case ε = 0.9, the friction factor and the overall heat transfer coefficient increases by about 31% and 27%, respectively, at $Re_{p}$~100. At $Re_{p}$~5000, the corresponding values are 59% and 26%. In comparison to cell size, it can be inferred that the change in porosity imparts greater influence on heat transport, mainly at a lower flow rate. As per Equation (17), the effective foam conductivity is regulated by the porosity term in such a way that the contribution of bulk foam conductivity increases upon lowering the porosity, which in turn increases the overall conductivity. Since the conductive heat transfer mechanism is dominant at lower flow rates (see Section 3.4), the heat transfer performance increases under such conditions at a reduced porosity.

The amount of fluid flowing through the pellets is shown in Figure 6c. It is revealed that the average amount of flow through the pellets is relatively low at $Re_{p}$~100, and the peak is about 12% for ε = 0.95, whereas the internal flow increases to about 35% at $Re_{p}$~5000. Also, the amount of fluid streams through the pellets shows little variation for porosities > 0.80 at constant $Re_{p}$ and cell size. According to Equation (14), the porosity term holds a power factor of 3, which indicates the strong influence of porosity on the pellet’s flow resistance. When the flow velocity is higher, the incoming flow has sufficient momentum to overcome the resistance induced by the pellets, consequently increasing the intra-particle flow.

Figure 6d shows the scalar plot of normalized velocity along a cross-sectional plane (P1) at $Re_{p}$~5000 for porosities 0.55 and 0.95. The reduction in porosity causes increased flow resistance inside the pellets, thereby diverting the flow around the particles, i.e., along the interstitial voids. This results in an increased pressure drop, yet favors the radial heat transfer. As mentioned earlier, the lateral fluid-mixing is intensified when flow accelerates along the interstitial voids, which in turn acts as the main driver in transferring the heat from the reactor wall towards the bed interior or vice versa. Similar to the cell size, the nature of the porosity property also contrasts with the design requirements of the reactor—lower pressure drop and improved heat transfer.

### 3.4. Effect of Conductivity

**Figure 7:**
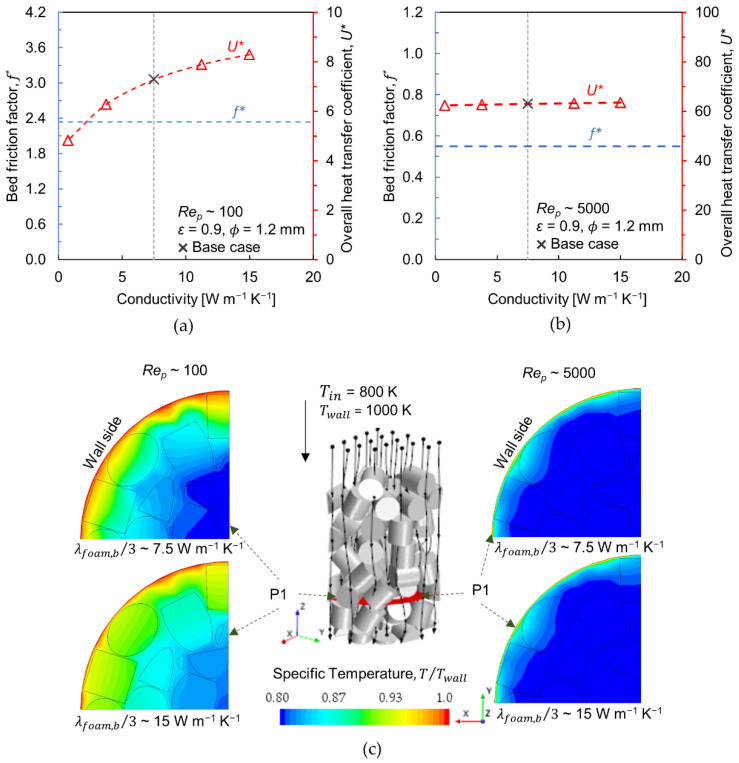
(**a**,**b**) Bed friction factor (left *y*-axis, circle markers) and overall heat transfer coefficient (right *y*-axis, triangle markers) versus foam conductivity at *Re_p_*~100 and 5000, respectively (base case is $1/3\cdot \lambda_{foam,b}$—Equation (18); (**c**) Normalized temperature contours at *Re_p_*~100 (left) and *Re_p_*~5000 (right) for conductivities 0.75 W m^−1^ K^−1^ and 15 W m^−1^ K^−1^ (P1 is the cross-sectional plane where the velocity contours are shown, stream lines through the bed are represented by black lines with arrow heads).

The latest manufacturing techniques have the capability to tune the material properties, for example, different alloy types and compositions that could improve the thermal conductivity [8]. The influence of foam conductivity on radial heat transfer has been investigated by assuming different levels of conductivity. Here, one-third of foam bulk conductivity proved by Equation (18) is considered as the base case (7.5 W m^−1^ K^−1^), and a conductivity level of double the base cases is regarded as an upper limit (15 W m^−1^ K^−1^), with 10% base case as a lower limit (0.75 W m^−1^ K^−1^). To identify the influence of foam conductivity explicitly, the structural parameters are kept constant–ϕ = 1.2 mm, ε = 0.9. Figure 7a,b show the overall heat transfer coefficient at $Re_{p}$~100 and $Re_{p}$~5000, respectively. It is revealed that the change in solid conductivity has negligible impact on radial heat transport at $Re_{p}$~5000. However, at $Re_{p}$~100, the heat transfer coefficient drops by about 34% when used at the lower conductivity level (10% of the base case) and increases by about 14% upon doubling the conductivity level relative to the base case.

The radial heat transport in a packed bed reactor can be represented by the effective radial conductivity $\Lambda_{er}$, which is a lumped parameter that sums up all radial heat transfer mechanisms taking place within the bed interior. The corresponding correlation is [43]:

(20) $$\frac{\Lambda_{er}}{\lambda_{f}}=\frac{\lambda_{b_{ed}}}{\lambda_{f}}+\frac{P_{e_{p}}}{{Κ}_{r}}$$

The first summand in Equation (20) represents pure conduction, where $\lambda_{bed}$ is the stagnant bed conductivity and $\lambda_{f}$ is the fluid conductivity. The second summand denotes radial heat transport occurring due to lateral-mixing of the fluid in the bed interstitial spaces, where $Pe_{p}=v_{s}{\left(\rho C_{p}\right)}_{f}d_{p,v}/\lambda_{f}$ is the molecular Peclet number; ρ and $C_{p}$ are the density and specific heat of the fluid. The intensity of mixing is represented by a limiting parameter ${Κ}_{r}$, which depends solely on the particle shape when the packed bed is of an infinite extent, i.e., negligible wall effects in radial void distribution. For such a packed bed composed of non-porous spherical particles ${Κ}_{r}$ = ~8 and with cylinders ${Κ}_{r}$ = ~4.6 [44]. It can be perceived from Equation (20) that $\Lambda_{er}\approx \lambda_{bed}$ at low flow rates or low $Pe_{p}$, whereas $\Lambda_{er}\approx Pe_{p}/{Κ}_{r}$ for high flow rates. Thus, the conductive heat transfer mechanism has a greater role at very low flow rates, however, the convective type of energy transport is dominant at higher flow velocities. This indicates that the thermal conductivity of catalyst pellets could not significantly influence an overall heat transfer performance of a packed bed reactor at high flow rates. Figure 7c shows the variation in radial temperature distribution along a cross-sectional plane (P1) for different conductivity levels at $Re_{p}$~100 (left plots) and ~5000 (right plots). The influence of pellet conductivity is distinguishable only at $Re_{p}\sim 100$, where the change in conductivity is reflected in the dominant heat transfer mechanism, i.e., conduction.

### 3.5. Design Study

The efficiency of a packed bed reactor is characterized by its low-pressure drop capability and high heat transport. In fact, these design requirements are found to contradict each other based on foam morphological parameters. As discussed earlier, an increase in cell size and porosity favors the reduction in pressure drop, however, it causes a drop in heat transfer efficiency too. Furthermore, regulating radial heat transfer by material properties is challenging, especially at very high flow rates, where the convective heat transfer mechanism plays a major role. There is no objection to the fact that using foam pellets would significantly reduce pressure drop due to the flow through pellets. At the same time, a certain level of fluid mixing along interstitial voids is also important to enhance the heat transfer between the reactor wall and the bed interior. Therefore, a suitable combination of foam structural parameters should be selected for achieving a reasonable trade-off between pressure drop and heat transfer efficiency.

An optimization study was carried out using the Design Manager utility provided by Siemens Simcenter STAR-CCM+, in which different combinations of cell size and porosity, called design sets, have been examined and for each design set a performance rating (PR) is determined as [29]:

(21) $$\mathrm{PR}=\sum_{i=1}^{N_{obj}}W_{i}S_{i}Obj_{i}-\sum_{j=1}^{N_{con}}\mathrm{Quad}W_{j}\cdot \mathrm{Con}Viol_{j}$$

where: $N_{obj}$ is the number of objectives, $W_{i}$ is the linear weight assigned to *i*-th objective,

$S_{i}$ is the sign for the *i*-th objective with a value of −1 for minimizing and +1 for maximizing, $Obj_{i}$ is the response value for the *i*-th objective, $N_{con}$ is the number of constraints, $QuadW_{i}$ is the quadratic weight of the *j*-th constraint, $ConViol_{j}$ is the amount by which the *j*-th constraint is violated.

**Figure 8:**
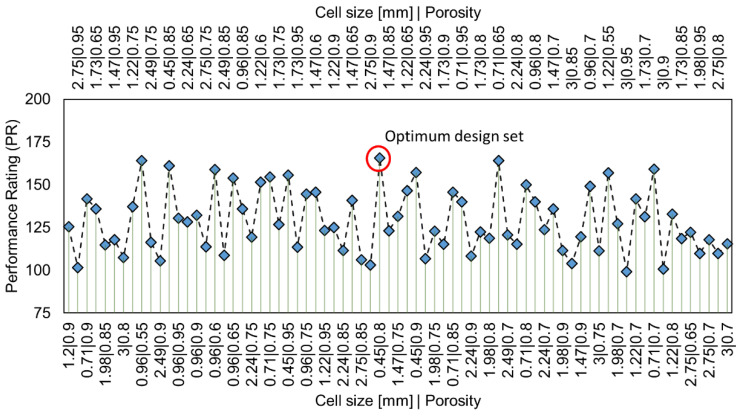
Performance Rating at *Re_p_*~5000 (two horizontal axes, on top and bottom, denote design sets as combination of cell size and porosity, optimum design set yields maximum PR: 𝜙 = 0.45 mm, *ε* = 0.8).

A total of 96 design sets were considered, with the objective to minimize friction and maximize the overall heat transfer coefficient. The heat transfer coefficient is given a weightage of 2, as maintaining an appropriate bed temperature level is essential for effective catalytic reactions. Additionally, a constraint is set such that the value of friction factor cannot exceed one, which guarantees sufficient reduction in pressure drop as compared to conventional packed beds, i.e., with solid particles. Figure 8 shows the computed PR for different design sets at $Re_{p}\sim 5000$. The corresponding friction factors and overall heat transfer coefficients are provided in Table A1-Appendix A.

The design set (ϕ = 0.45 mm, ε = 0.8) has achieved the maximum PR = 165.66. The other viable design sets are: [(0.96 mm, 0.55, PR = 164.09), (0.71 mm, 0.65, PR = 164)]. From the manufacturing perspective, it might be impractical to produce any combination of ϕ and ε, as it is dependent on production methods. Hence, the feasibility of manufacturing should also be considered in the selection of optimum foam parameters. Furthermore, the specific surface area is an important property for catalytic reactions. As provided by the manufacturer (Alantum Europe GmbH), ϕ=0.45 mm, ε=0.8 bears a specific surface area of about 9040 m^2^/m^3^, and ϕ=1.2 mm, ε=0.9 holds about 4320 m^2^/m^3^. Thus, a small cell size and a medium porosity are a better choice, as they provide high surface area and a good compromise between pressure drop and heat transfer.

It is also inferred that a significant change in heat transfer efficiency is difficult to achieve by adjusting only the foam structural properties, as the observed standard deviation of overall heat transfer coefficient is only about 9 upon 73 successful design sets (see Table A1, Appendix A). Another option to improve the heat transfer performance is by modifying the shape of foam pellets along with the optimum structural parameters. In this regard, hollow ring-like shapes might be a better choice, as they provide more flow diversions by allowing the fluid through the inner holes and around them, subsequently enhancing lateral mixing of the fluid and therefore intensifying radial heat transport.

### 3.6. Correlations for Friction Factor and Heat Transfer Coefficient

The correlations for the prediction of transport quantities in a foam-packed bed with different foam morphologies have been derived by using CFD data. The geometric features of the foam and the flow regime are indicated by particle Reynolds number $Re_{p}$ are combined with appropriate fitting constants to match with the observed data.

The derived correlation for the friction factor $f^{*}$ is:

(22) $$f^{*}=\underset{Ergun\;modified}{\underbrace{\left[\frac{150}{{\left(Re_{p}/1-\xi \right)}^{r^{\prime }}}+1.75\right]}}-\left[{\left(\frac{\phi \left[m\right]}{0.001m}\right)}^{m^{\prime }}{\left(1-\varepsilon \right)}^{n^{\prime }}\right]$$

with:

(23) $$\begin{array}{l} r^{\prime }=-0.01790F_{g}^{2}+0.13451F_{g}+0.74664;\;F_{g}=\left(\frac{\phi \left[m\right]}{0.001m}\cdot \varepsilon \right)\\ \;\;m^{\prime }=0.23,\;n^{\prime }=-0.057 \end{array}$$

Here, ξ is the mean interstitial bed voidage. The first term in Equation (22) is an adapted form of the friction factor provided by the Ergun equation for a conventional packed bed system, i.e., solid particles. The modification is in $Re_{p}$ by including a power factor $r^{\prime }$, which accounts for the intra-particle flow. Thus, $r^{\prime }$ is dependent on foam morphology and an expression has been formulated to determine $r^{\prime }$ in terms of ϕ and ε, see Equation (23). For example, $r^{\prime }=0.98$ for ϕ = 3 mm and ε = 0.9 while $r^{\prime }$ = 0.8 for ϕ = 0.45 mm. The non-linear dependency of friction factor with $Re_{p}$ in foam-packed beds has been reported also in ref. [42]. The term $\frac{\phi \left[m\right]}{0.001m}$ is coined by referring Incera Garrido et al. [12], where a similar type of nondimensionalization has been introduced with the foam pore diameter for deriving a mass transfer correlation in foam monoliths. As they have reported, this term is not based on any physical grounds and is rather formulated by fitting the transport data, thereby avoiding the inclusion of complex parameters to represent the fine foam geometries in the correlation, making it simple for the user. It should be noted that ‘m’ in $\frac{\phi \left[m\right]}{0.001m}$ denotes meter.

**Figure 9:**
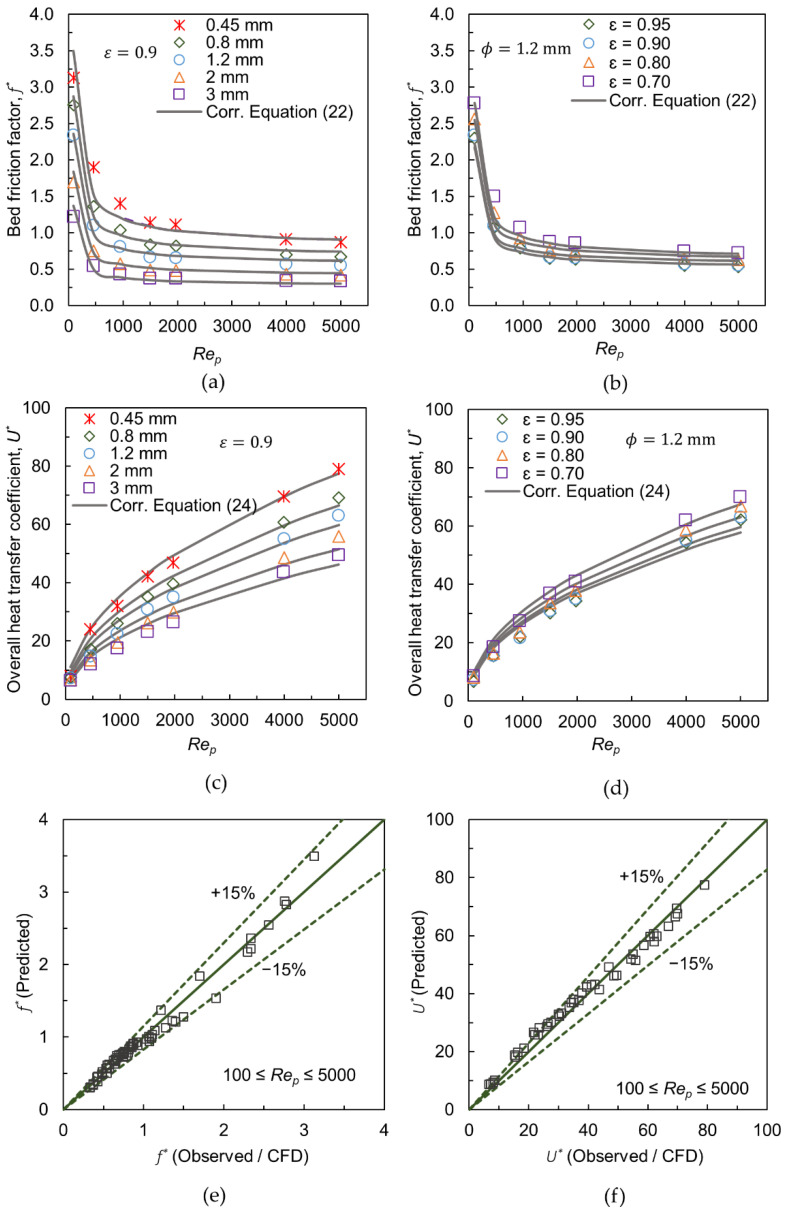
(**a**,**b**) Comparison of friction factors with correlation Equation (22) for different cell sizes and porosities, respectively; (**c**,**d**) Comparison of overall heat transfer coefficients with correlation Equation (24) for different cell sizes and porosities, respectively; (**e**,**f**) Parity plots for friction factor and overall heat transfer coefficient, respectively.

Figure 9a shows the comparison of friction factors predicted by correlation with observed data for different cell sizes and ε = 0.9 over $\sim 100\leq Re_{p}\leq \sim 5000$. The agreement is good for the cases investigated, except for minor differences for ϕ = 0.45 and low $Re_{p}$. When the cell size is reduced, the foam particles exhibit greater resistance to internal flow, which causes a jump in friction factor, mainly at lower velocities. Figure 9b depicts the friction factor predicted by the correlation for ε=0.7−0.95 with ϕ=1.2 mm. The comparison with observed data also indicates a good agreement. Figure 9e shows the parity plot, which reveals that the proposed correlation Equation (22) is capable to predict the friction factor with respect to foam structural parameters: 0.45 mm≤ϕ≤1.2 mm and 0.70≤ε≤0.95, over $\sim 100\leq Re_{p}\leq \sim 5000$ with a reasonable accuracy of ±15%.

The correlation for the overall heat transfer coefficient Equation (19), developed for the cylindrical foam pellets with ϕ=1.2 mm and ε = 0.9, is extended to account for the different values of structural parameters as:

(24) $$U^{*}=0.586\cdot Re_{p}^{0.486}{\left(\frac{\phi \left[m\right]}{0.001m}\right)}^{m^{\prime }}\left(a^{\prime }\cdot \varepsilon^{n^{\prime }}\right)$$

with:

(25) $$\begin{array}{l} a^{\prime }=0.0231F_{g}^{2}-0.1327F_{g}+1.7704;\;F_{g}=\left(\frac{\phi \left[m\right]}{0.001m}\cdot \varepsilon \right)\\ \;\;\;m^{\prime }=-0.235,\;n^{\prime }=-0.257 \end{array}$$

Figure 9c shows the comparison of the overall heat transfer coefficient predicted by the correlation with observed data for different cell sizes and ε = 0.9. The agreement is satisfactory for the investigated $Re_{p}$ range. In the range of low $Re_{p}<500$, the overall heat transfer coefficient increases almost linearly, where the conductive heat transfer mechanism plays a major role, and intra-particle flow is low. At higher $Re_{p}$, the flow through foam pellets increases, and the convective mechanism takes the primary role in heat transfer. Since a significant intra-particle flow hinders the lateral fluid-mixing, the convective mode of heat transfer is less intense in a foam-packed bed than in a conventional packed bed. Due to this, the overall heat coefficient shows a half-parabolic profile, when $Re_{p}$ increases. Figure 9d denotes that the correlation is also comparable with observed data for the cases of different porosities and ϕ = 1.2 mm. As indicated by the parity plot in Figure 9f, this correlation does provide reasonable predictions ±15%, with the exception of a few outliers at low Reynolds numbers.

In summary, the proposed correlations could aid engineers in determining the important transport quantities for different foam geometric features in the simplest way.

## 4. Conclusions

A wall-segment CFD setup was used to investigate the effect of foam morphologies on the transport processes in a randomly packed bed composed of cylindrical open-cell metallic foam pellets. The modeling strategy is in the framework of particle-resolved CFD with sub-models to mimic flow and heat transfer inside the foam pellets. Instead of resolving the inner geometries of foam pellets, the corresponding pressure loss and effective thermal conductivity were modeled by the porous-media approach at the pellet level. The flow and energy transport in interstitial voids are considered as they are, which is important in capturing the local bed structural effects in such a slender packed bed system. The Rigid Body Dynamics method was used to generate the packing structure.

The use of wall-segment CFD has been found worthy for the parametric analysis, since a significant reduction in computational time is achieved even using a normal computing system, i.e., full-bed simulations required several hours or day with 1 CPU—intel Core i7-8700K were instead carried out under half-hour.

The main findings from the parametric analysis covered in the present work are:

- The friction factor and the overall heat transfer coefficient decrease with an increase in cell size and porosity.
- The observed behavior contradicts the desired requirements in a packed bed, i.e., lower pressure drop and higher heat transfer.
- The transport behavior in a foam-packed bed is dependent on the amount of flow stream through the pellets, which is regulated by the flow velocity in addition to foam morphologies.
- The intra-particle flow increases by increasing the cell size and porosity added with higher flow velocity.
- The influence of the conductivity of foam pellets on the overall heat transfer of a packed bed is found to be negligible at higher flow rates; the convective heat transfer mechanism is dominant in such conditions, which can be influenced most significantly by the pellet shape and dimensions.
- Foam morphologies, as well as shape, should be optimized to achieve a trade-off between pressure drop and heat transfer efficiency.
- A design study has shown that a cell size of 0.45 mm and a porosity of 0.80 is the optimal foam morphology of a cylindrical foam pellet for *Re_p_*~5000.

Based on CFD data, suitable correlations for predicting the friction factor and the overall heat transfer coefficient that accounts for different foam morphologies have been derived for $100\leq Re_{p}\leq 5000$, with an accuracy of ±15%.

Further research is recommended to investigate different foam pellet shapes and tube-to-particle ratios. It would also be useful to conduct reactive simulations to investigate the influence of foam morphologies on conversion rates and temperature distribution within the bed.

## Figures and Tables

**Table 1:** Basic properties of foam pellet.

Pellet	$d_{p,v}\;[\mathrm{mm}]$	^1^ $N=D/d_{p}$	ϕ [mm]	ε	^2^ $\lambda_{p}$ $\left[W\;m{}^{-1}\;K{}^{-1}\right]$	*C*_*p*_ [J kg^−1^ K^−1^]	^3^ $\rho_{p}$ $\left[\mathrm{kg}\;m{}^{-3}\right]$
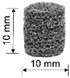	11.45	6.78	1.2 ± 0.12	0.90 ± 0.02	0.19 ± 0.003	580	650

^1^ tube-to-particle diameter ratio, D = 67.8 mm. ^2^ effective stagnant conductivity at room temperature. ^3^ apparent density.

**Table 2:** CFD simulation inputs (pressure, temperature, feed gas from ref. [40,41]).

Inlet Velocity, *v_s_* [m s^−1^]	0.032 and 1.62
Particle Reynolds number, *Re_p_*	~100 and ~5000
Feed compositions (in mole fraction):	
Steam	0.7485
CH_4_	0.2143
CO_2_	0.0025
N_2_	0.0347
Inlet temperature, *T*_in_ [K]	800
Wall temperature, *T_wall_* [K]	1000
Total pressure [bar]	29

## Data Availability

The data presented in this study are available on request from the corresponding author.

## Appendix A

**Table A1:** Successful Design sets.

Design	Cell Size [mm]	Porosity	*f**	*U**	PR
1	1.20	0.90	0.550	62.980	125.411
2	2.75	0.95	0.334	50.916	101.499
3	0.71	0.90	0.710	71.261	141.812
4	1.73	0.65	0.662	68.289	135.916
5	1.98	0.85	0.455	57.640	114.825
6	1.47	0.95	0.478	59.212	117.946
7	3.00	0.80	0.395	53.934	107.473
8	1.22	0.75	0.668	68.943	137.217
9	0.96	0.55	0.952	82.523	164.093
10	2.49	0.75	0.476	58.451	116.427
11	2.49	0.90	0.370	52.923	105.476
12	0.45	0.85	0.910	81.035	161.161
13	0.96	0.95	0.596	65.472	130.349
14	2.24	0.65	0.592	64.430	128.268
15	0.96	0.90	0.614	66.352	132.09
16	2.75	0.75	0.453	57.101	113.749
17	0.96	0.60	0.892	79.938	158.984
18	2.49	0.85	0.401	54.551	108.701
19	0.96	0.65	0.837	77.374	153.911
20	0.96	0.85	0.652	68.222	135.793
21	2.24	0.75	0.503	59.950	119.398
22	1.22	0.60	0.815	76.196	151.577
23	0.71	0.75	0.841	77.659	154.478
24	1.73	0.75	0.570	63.648	126.725
25	0.45	0.95	0.851	78.207	155.563
26	1.73	0.95	0.438	56.997	113.557
27	0.96	0.75	0.739	72.650	144.56
28	1.47	0.60	0.759	73.239	145.719
29	1.22	0.95	0.528	61.940	123.352
30	1.22	0.90	0.546	62.796	125.046
31	2.24	0.85	0.426	55.992	111.559
32	1.47	0.65	0.708	70.734	140.761
33	2.75	0.85	0.379	53.270	106.161
34	2.75	0.90	0.349	51.672	102.994
35	0.45	0.80	0.961	83.312	165.664
36	1.47	0.85	0.530	61.826	123.121
37	1.47	0.75	0.614	66.031	131.448
38	1.22	0.65	0.763	73.667	146.571
39	0.45	0.90	0.868	79.022	157.176
40	2.24	0.95	0.378	53.545	106.712
41	1.98	0.75	0.534	61.660	122.786
42	1.73	0.90	0.455	57.822	115.19
43	0.71	0.85	0.749	73.210	145.672
44	0.71	0.95	0.693	70.384	140.076
45	2.24	0.90	0.394	54.334	108.274
46	1.73	0.80	0.528	61.527	122.526
47	1.98	0.80	0.493	59.584	118.675
48	0.71	0.65	0.946	82.473	163.999
49	2.49	0.70	0.518	60.585	120.651
50	2.24	0.80	0.463	57.904	115.345
51	0.71	0.80	0.793	75.370	149.946
52	0.96	0.80	0.694	70.389	140.083
53	2.24	0.70	0.546	62.118	123.691
54	1.47	0.70	0.659	68.321	135.983
55	1.98	0.90	0.422	55.944	111.466
56	3.00	0.85	0.360	52.118	103.876
57	1.47	0.90	0.495	60.061	119.626
58	0.96	0.70	0.787	74.970	149.154
59	3.00	0.75	0.432	55.895	111.358
60	1.22	0.55	0.872	78.927	156.982
61	1.98	0.70	0.577	63.907	127.237
62	3.00	0.95	0.316	49.803	99.2887
63	1.22	0.70	0.715	71.210	141.706
64	1.73	0.70	0.615	65.915	131.214
65	0.71	0.70	0.891	80.017	159.143
66	3.00	0.90	0.331	50.547	100.763
67	1.22	0.80	0.624	66.723	132.822
68	1.73	0.85	0.489	59.553	118.616
69	2.75	0.65	0.540	61.407	122.275
70	1.98	0.95	0.405	55.136	109.867
71	2.75	0.70	0.494	59.219	117.944
72	2.75	0.80	0.415	55.110	109.806
73	3.00	0.70	0.473	57.987	115.501

## Nomenclature

Latin symbols		
$A^{\prime }$	area	m^2^
a	pore diameter	m
$d_{p}$	particle diameter	m
$d_{p,v}$	diameter of sphere of equivalent particle volume	m
$d_{s}$	strut diameter	m
D	tube diameter	m
$f^{*}$	friction factor	
L	bed length	m
ΔP	pressure drop	Pa
$Pe_{p}$	Peclet number	
$Re_{p}$	particle Reynolds number	
T	temperature	K
U	overall heat transfer coefficient	W m^−2^ K^−1^
$U^{*}$	normalized heat transfer coefficient	
$v_{s}$	superficial velocity	m s^−1^
Greek symbols		
ε	foam porosity	
λ	thermal conductivity	W m^−1^ K^−1^
$\Lambda_{er}$	effective radial conductivity	W m^−1^ K^−1^
μ	dynamic viscosity	Pa s
ξ	mean bed voidage	
ρ	density	kg m^−3^
ϕ	foam cell size	m
${Κ}_{r}$	limiting value	
